# The American and Colonial Journals: Pathology, Practical Medicine, and Therapeutics

**Published:** 1842-10

**Authors:** 


### PATHOLOGY, PRACTICAL MEDICINE, AND THERAPEUTICS.

*Chronic Pleuritis, with Ossification of the Diaphragm.* By James Pagan, Esq.

Rohun, a convict, was sickly, emaciated, and unable to labour, although his tongue, skin, &c., were quite natural, and his appetite good. He could only speak in a gentle whisper. It appeared on inquiry at the native doctor, that the man had been often in the hospital during the last two years, with fever; but no mention was made of thoracic disease; although, on examination, the lower part of the right side of the chest was found to be dull. On inspection, there was found close to the sternum, a perfect cavity formed by a strong false membrane, which contained from four to six ounces of serum. The inner coat of this cavity was soft and jelly-like; but the outer was strong and thick. There were strong adhesions between the costal and pulmonary pluræ, and the right lung was considerably hepatized.

The portion of diaphragm, situated between the liver and right lung, was ossified to a considerable extent, so much so that it was difficult to remove or cut it with a knife.

[We have again to call attention to the deplorable typography of this Journal, and to object to the constant practice of transferring entire articles from our pages, without the civility of acknowledgment. The article on Bonnet and Thomson on Hepatic Disease, in No. 73, is a *verbatim et literatim* transcription from our columns, without the slightest indication as to the source from which it is obtained. We must also express our disappointment at the extreme paucity of original contributions in this periodical, which will account for the small number of "Selections" which we are enabled to make from it.]

*India Journal of Medical and Physical Science.* No. lxxi.. *Nov.* 1, 1841.

### MIDWIFERY, AND DISEASES OF WOMEN AND CHILDREN.

